# Food Safety: Perchlorate Exposure: Tip of the Iceberg?

**Published:** 2005-04

**Authors:** Rebecca Renner

For several years, federal and state agencies have debated over what is an acceptable level of human perchlorate exposure through food and drinking water. Now Food and Drug Administration (FDA) investigators have found the chemical in milk and lettuce from 15 states, including some apparently uncontaminated areas, showing that human exposure may come from more sources than expected.

Perchlorate is used mainly in rocket fuel as well as in some fertilizers and explosives. Perchlorate with no anthropogenic source has been found at 20–60 parts per billion (ppb) in West Texas groundwater and in trace amounts in precipitation, says Texas Tech University chemist Purnendu Dasgupta. This suggests atmospheric reactions may create a low background level of perchlorate. Perchlorate disrupts thyroid function by competitively inhibiting iodine uptake in a dose-dependent fashion, with unquantified effects in humans.

In a November 2004 agency report, FDA scientists wrote of finding an average 7.76–11.9 ppb perchlorate in about 90% of lettuce samples from Arizona, California, Florida, New Jersey, and Texas. They also found an average of 5.76 ppb in 97% of cow’s milk samples collected at stores in 14 states. Until more is known about the health effects of perchlorate and its occurrence in foods, the FDA continues to recommend that people of all ages eat a balanced, healthy diet.

Parts of southern Arizona and California are irrigated with river water containing roughly 4–6 ppb perchlorate, but contamination is not known at the other sites. “The results are surprising—we would have expected lettuce grown in known perchlorate-contaminated areas to have higher concentrations than lettuce from apparently uncontaminated areas,” says Terry Troxell, director of the FDA Office of Plant and Dairy Foods. Troxell says samples with very high and very low values came from the same place. For example, the highest lettuce concentration was 71.6 ppb in iceberg lettuce from Belle Glade, Florida. But another Belle Glade iceberg sample contained 1.3 ppb.

“I don’t think it’s possible to conclude anything about the national food supply from this survey,” says Kevin Mayer, the Environmental Protection Agency Region 9 perchlorate coordinator. Still, says Bill Walker, West Coast director for the nonprofit Environmental Working Group, “The surprising data suggest that this is a national problem and that risk assessments have to account for dietary exposure.”

In January 2005 the National Academy of Sciences reported that more information is needed on food as a source of perchlorate exposure. Meanwhile, the evidence rolls in. In the 26 January 2005 *Journal of Agricultural and Food Chemistry*, Texas Tech researchers reported finding perchlorate in a variety of forage and edible crops, including alfalfa and cantaloupe. The FDA is also sampling tomatoes, carrots, cantaloupe, and spinach, with results to come.

## Figures and Tables

**Figure:**
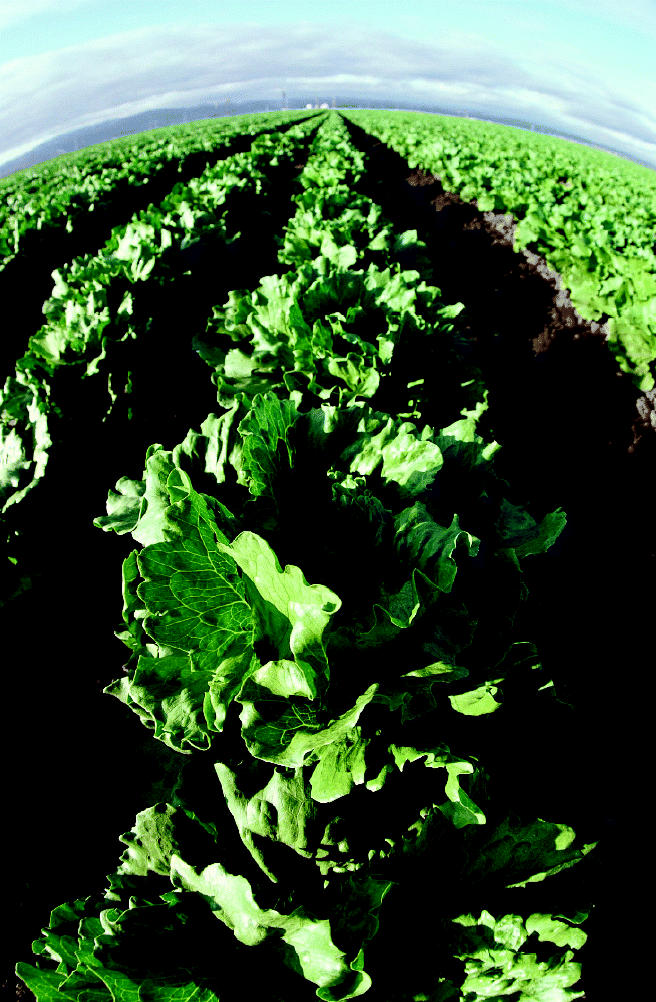
**Salad surprise.** Perchlorate has turned up in foods including lettuce samples from five states.

